# Astrocytic and Neuronal Apolipoprotein E Isoforms Differentially Affect Neuronal Excitability

**DOI:** 10.3389/fnins.2021.734001

**Published:** 2021-09-21

**Authors:** Sabine C. Konings, Laura Torres-Garcia, Isak Martinsson, Gunnar K. Gouras

**Affiliations:** Experimental Dementia Research Unit, Department of Experimental Medical Science, Lund University, Lund, Sweden

**Keywords:** Apolipoprotein E, Alzheimer’s disease, synapse, neuron, astrocyte, amyloid, calcium imaging, hyperexcitability

## Abstract

Synaptic changes and neuronal network dysfunction are among the earliest changes in Alzheimer’s disease (AD). Apolipoprotein E4 (ApoE4), the major genetic risk factor in AD, has been shown to be present at synapses and to induce hyperexcitability in mouse knock-in brain regions vulnerable to AD. ApoE in the brain is mainly generated by astrocytes, however, neurons can also produce ApoE under stress conditions such as aging. The potential synaptic function(s) of ApoE and whether the cellular source of ApoE might affect neuronal excitability remain poorly understood. Therefore, the aim of this study was to elucidate the synaptic localization and effects on neuronal activity of the two main human ApoE isoforms from different cellular sources in control and AD-like *in vitro* cultured neuron models. In this study ApoE is seen to localize at or near to synaptic terminals. Additionally, we detected a cellular source-specific effect of ApoE isoforms on neuronal activity measured by live cell Ca^2+^ imaging. Neuronal activity increases after acute but not long-term administration of ApoE4 astrocyte medium. In contrast, ApoE expressed by neurons appears to induce the highest neuronal firing rate in the presence of ApoE3, rather than ApoE4. Moreover, increased neuronal activity in APP/PS1 AD transgenic compared to wild-type neurons is seen in the absence of astrocytic ApoE and the presence of astrocytic ApoE4, but not ApoE3. In summary, ApoE can target synapses and differentially induce changes in neuronal activity depending on whether ApoE is produced by astrocytes or neurons. Astrocytic ApoE induces the strongest neuronal firing with ApoE4, while the most active and efficient neuronal activity induced by neuronal ApoE is caused by ApoE3. ApoE isoforms also differentially affect neuronal activity in AD transgenic compared to wild-type neurons.

## Introduction

Alzheimer’s disease (AD), the most common form of dementia, clinically manifests with cognitive and memory decline. On a neuropathological level, AD is characterized by the presence of amyloid plaques, consisting of aggregated β-amyloid (Aβ), and neurofibrillary tangles, containing abnormal hyperphosphorylated and aggregated tau. Apolipoprotein E4 (ApoE4) genotype is the major genetic risk factor for AD. In the brain ApoE is mainly produced by astrocytes. However, it is also generated by microglia and under stress ApoE can be produced by neurons (Boschert et al., 1999). Although astrocytic ApoE is the main source of ApoE in the brain, ApoE seems to play a role in many cell types and pathways related to AD (Liu et al., 2013; Lin et al., 2018). Currently, it remains unclear which cellular source of ApoE and pathological molecular mechanisms are the most critical for AD.

Many of the early cellular changes detected in AD have been associated with neurons, such as synaptic and endosomal alterations, intraneuronal Aβ accumulation and neuronal network dysfunction. In particular synaptic terminals seem to be affected in early stages of AD and synaptic dysfunction and loss are highly correlated with cognitive deficits in AD (DeKosky and Scheff, 1990; Scheff et al., 2006). In addition, hippocampal hyperactivity appears predictive of cognitive decline in mild cognitive impairment (MCI) patients and shows a negative correlation with neurodegeneration (Miller et al., 2008; Putcha et al., 2011). Both of the AD-linked proteins, Aβ and tau, have been associated with impaired synaptic network activation, where Aβ was shown to induce hyperexcitability, while tau has shown conflicting results, both enhancing and inhibiting network activity in the cortex of mice (Crimins et al., 2011; Angulo et al., 2017; Busche et al., 2019). Increased brain activity in regions vulnerable to AD, such as hippocampus and entorhinal cortex (EC), has been reported in MCI patients or carriers of familial AD mutations before the onset of AD (Miller et al., 2008; Quiroz et al., 2010). In addition, transgenic AD mice show neuronal hyperexcitability in AD vulnerable brain regions already before plaque onset (Palop et al., 2007; Busche et al., 2012).

ApoE4 seems to be involved in early neuronal and synaptic changes associated with AD. ApoE4 induces impaired endosome recycling, resulting in trapping of receptors involved in synaptic plasticity and ApoE-related lipid transport into endosomes causing dysregulated long-term potentiation (LTP) (Chen et al., 2010). Additionally, ApoE4 target replacement mice, devoid of amyloid pathology, show neuronal hyperexcitability in entorhinal cortex (Nuriel et al., 2017), a dysregulation also seen in AD transgenic mice and in early AD (Busche et al., 2012; Vossel et al., 2013). The specific role of ApoE4 in neurons has been further supported by a study showing that deletion of ApoE4 production selectively in GABAergic interneurons is sufficient to prevent GABAergic interneuron loss and learning and memory deficits in ApoE4 knock-in (KI) mice, while a deletion of astrocyte-produced ApoE4 was not protective (Knoferle et al., 2014).

Thus, despite astrocytes normally producing the majority of ApoE in the brain, the most crucial cellular source of ApoE in AD remains unclear and ApoE appears to have important effects on neuronal excitability and synaptic changes associated with AD. ApoE has previously been reported to be present at synaptic terminals (Koffie et al., 2012; Bilousova et al., 2019). Yet, the potential role of ApoE in neurons under both healthy and AD conditions remains poorly understood. The aim of this study was to elucidate the role(s) of ApoE isoforms on synapses and neuronal activity *in vitro* both in healthy and AD-like conditions of elevated Aβ. Different cellular sources of ApoE, from astrocytes and neurons, were studied to investigate whether the ApoE4-induced increased neuronal excitability as reported *in vivo* might depend on its cellular origin.

## Materials and Methods

### Antibodies and Reagents

The following antibodies were used: ApoE (Invitrogen, 16H22L18), Western blot (WB): 1:250, immunofluorescence (IF): 1:500; ApoE (WUE-4) (Novus Biologicals, NB110-60531), WB: 1:500; β-actin (Sigma-Aldrich, A5316), WB: 1:2000; Ca2+/calmodulin-dependent protein kinase II (CaMKIIα) (Sigma-Aldrich, 05-532), IF: 1:500; 4′,6-diamidino-2-phenylindole (DAPI) (Sigma-Aldrich, D9542), IF: 1:2000; microtubule associated protein 2 (MAP2) (Abcam, ab92434), IF: 1:1000; rhodamine phalloidin (Invitrogen, R415), IF: 1:2000; vesicular GABA transporter (VGAT) (Synaptic Systems, 131013), IF: 1:500; vesicular glutamate transporter 1 (vGlut1) (Sigma-Aldrich, ab5905), IF: 1:500.

Secondary antibodies Alexa Fluo^®^ 488 AffiniPure Goat Anti-Rabbit IgG (Jackson ImmunoResearch, 111-545-144), Alexa Fluo 568 Goat anti-Mouse IgG (Invitrogen, A-11004), Alexa Fluo 633 Goat anti-Guinea pig IgG (Invitrogen, A-21105), and Alexa Fluor^®^ 647 AffiniPure Donkey Anti-Chicken IgY (IgG) (Jackson ImmunoResearch, 703-605-155) (all 1:500) were used for immunofluorescence. HRP-conjugated rabbit IgG (R&D systems, HAF008) and mouse IgG (R&D systems, HAF007) antibodies (1:2000) were used as secondary antibodies for Western blot.

Recombinant ApoE3 (Sigma-Aldrich, SRP4696), used as a positive control for ApoE on Western blot, was reconstituted in 0.1% bovine serum albumin (BSA) (Sigma-Aldrich, A7906) in milli-Q water to a stock concentration of 0.1 mg/ml.

### Ethical Considerations

All experiments using animals in this study were approved by the Ethical Committee for animal research at Lund University under ethical permit M5983-19. The ethical permission allows the use of brain cells from embryonic and neonatal mice for scientific research. Mice used for this study were ApoE3 KI (B6.Cg-Apoeem2(APOE^∗^)Adiuj/J), ApoE4 KI (B6(SJL)-Apoetm1.1(APOE^∗^4)Adiuj/J), ApoE KO (B6.129P2-Apoe < tm1Unc > /J), and APP/PS1 (B6.Cg-Tg (APPswe, PSEN1dE9)85Dbo/Mmjax) that were all purchased from Jackson Laboratory.

### Isolation and Culture of Primary Mouse Neurons

Cortical and hippocampal mouse primary neurons were obtained from embryonic (E15-E17) mouse brains. The protocol used to obtain mouse primary neurons was previously described by Takahashi et al. (2004). Embryonic cortical and hippocampal brain tissue was dissected and dissociated into single cells using 0.25% trypsin (Thermo Fisher Scientific, 15090046). Neurons were seeded on poly-D-lysine coated plates in Dulbecco’s modified Eagle medium (DMEM) with 10% fetal bovine serum (FBS) (Gibco, 10082147) and 1% penicillin/streptomycin (P/S) (Thermo Fisher Scientific, SV30010). 3–5 h after seeding, the medium was changed to Neurobasal medium containing B27 supplement (Gibco, 17504044), 1% P/S and 1.4 mM L-glutamine (Gibco, 25030081). Primary neurons were cultured for 18–20 days *in vitro* (DIV) before usage for either live cell imaging, western blot or immunofluorescence.

### Isolation and Culture of Primary Mouse Astrocytes

Primary mouse astrocytes were obtained from cortical and hippocampal tissue from ApoE KO, ApoE3 KI and ApoE4 KI pups (P1–P3). Mouse brains were dissected and incubated in 0.25% trypsin, followed by dissociation of the cells using soft plastic Pasteur pipets in DMEM containing 10%, FBS and 1% P/S. Astrocytes were plated on poly-D-lysine coated T75 plates at a concentration of 500,000 cells/plate and after 3–5 h the medium was replaced by AstroMACS medium (Miltenyi Biotec, 130-117-031) containing 0.5 mM L-glutamine. The medium of the primary astrocytes was changed every 2–3 days until the astrocytes reached at least 80% confluence.

### Neurons Cultured in Astrocyte Conditioned Media

Astrocyte conditioned medium was collected from ApoE KO, ApoE3 KI and ApoE4 KI primary astrocytes. Once primary astrocyte cultures reached 80% confluence, primary astrocytes cultured in AstroMACS medium were shortly washed in phosphate buffered saline (PBS) and then cultured in Neurobasal medium with B27 supplement, 1% P/S and 1.4 mM L-glutamine for 48 h. After 48 h, the astrocyte-Neurobasal conditioned medium was collected on ice, centrifuged at 10,000 × *g* for 10 min at 4°C and stored at −80°C until further use. Western blot was performed after each astrocyte conditioned media collection to confirm similar ApoE levels between different ApoE conditions. When adding conditioned astrocytic media to neurons, half of the original Neurobasal medium supplemented with B27 supplement, 1% P/S and 1.4 mM L-glutamine was removed from primary neuron cultures at 18–20 DIV and astrocyte-Neurobasal conditioned medium was added resulting in 50% astrocyte-Neurobasal conditioned media, 50% neuronal-Neurobasal conditioned media.

### Biochemical Analyses

#### SDS PAGE

ApoE protein expression was detected in neuronal lysate and astrocyte and neuron conditioned media by sodium dodecyl sulfate polyacrylamide gel electrophoresis (SDS PAGE) and Western blot. Medium was collected and centrifuged at 10,000 × *g* for 10 min at 4°C before use. Neuronal lysate was washed in PBS, homogenized/lysed in a buffer containing 20 mM Tris, 150 mM KCl, 5 mM MgCl_2_, and 1% NP40 with Halt^TM^ Protease inhibitor cocktail (Thermo Fisher Scientific, 78430) and Halt^TM^ Phosphatase Inhibitor Cocktail (Thermo Fisher Scientific, 78428), and centrifuged at 20,000 × *g* for 20 min at 4°C. The supernatant, which contained the intracellular proteins, was further used for Western blot. The media and lysate samples were mixed with Novex NuPage LDS sample buffer (Invitrogen, NP0007) and NuPage sample reducing agent (Invitrogen, NP0004), heated for 10 min at 70°C and loaded on a NuPAGE^TM^ 4–12% Bis-Tris protein gel (Invitrogen, NP0321). Secreted proteins in medium and intracellular proteins in lysate were separated by SDS PAGE. SeeBlue^®^ Plus2 pre-stained protein standard was used as a molecular weight marker.

#### Blue-Native PAGE

Non-denaturing Blue-Native PAGE was performed to investigate the native structure of ApoE complexes. Astrocyte and neuron media samples, and recombinant ApoE samples were mixed with NativePAGE^TM^ Sample Buffer (Invitrogen, BN2003). The samples were loaded onto a NativePAGE^TM^ 4–16% Bis-Tris gel (Invitrogen, BN1002BOX) and run at 150 V. NativePAGE running buffer (Invitrogen, BN2001) and NativePAGE^TM^ Cathode Buffer Additive (Invitrogen, BN2002) were used as running buffers. NativeMark^TM^ Unstained Protein Standard (ThermoFisher Scientific, LC0725) was run on the gel as molecular weight marker and was visualized, after protein transfer, using Ponceau C. After protein transfer, the membranes were boiled two times in PBS for 5 min, followed by 7 min incubation in 10% formic acid, prior to blocking, according to a protocol previously described by Heinsinger et al. (2016).

#### Western Blot

The samples were transferred to PDVF membranes using the iBlot^TM^ 2 Gel Transfer Device (Thermo Fisher Scientific, IB21001). The membranes were blocked in PBS containing 0.1% Tween (PBS-T) and 5% (for SDS PAGE) or 10% (for Blue-Native PAGE) non-fat dry milk (Millipore, 70166) and subsequently incubated with primary antibodies overnight at 4°C, followed by HRP-conjugated secondary antibodies for 1 h at room temperature. All washes were performed in PBS-T. The membrane was developed in Pierce^TM^ ECL Western Blotting Substrate (Thermo Fisher Scientific; 32106) and visualized using Sapphire Biomolecular imager (Azure Biosystems). Recombinant ApoE3 was added to each gel as a positive control for the ApoE antibody. ApoE protein levels in neuronal lysate on SDS gels were quantified using ImageJ 1.53c and normalized to loading control β-actin.

### Immunofluorescence and Microscopy

Mouse primary neurons were cultured on coverslips for 19 DIV before they were fixed in PBS containing 4% paraformaldehyde (PFA) and 4% sucrose for 15 min at room temperature. The neurons were permeabilized and blocked in PBS containing 0.1% saponin (Sigma-Aldrich, 84510), 1% BSA and 2% normal goat serum (NGS) (Jackson ImmunoResearch, 005-000-121) for 1 h at room temperature. In experiments to specifically study surface labeling of ApoE, neurons were not permeabilized by adding saponin and therefore only blocked in 1% BSA and 2% NGS in PBS. Subsequently the neurons were incubated with primary antibodies overnight at 4°C and after that incubated in fluorescently conjugated secondary antibodies for 1 h at room temperature in the dark. All primary and secondary antibodies were diluted in PBS containing 2% NGS. Neurons on coverslips were incubated with DAPI and/or phalloidin dyes for 5 or 30 min, respectively. After primary and secondary antibody incubations and incubation with DAPI or phalloidin, the coverslips were washed in PBS. The coverslips were mounted with ProLong^TM^ Diamond Antifade Mountant (Invitrogen, P36961).

The immunofluorescence signal in the neurons was imaged by epifluorescence microscopy or confocal microscopy. Epifluorescence microscopy was performed using an Olympus IX70 microscope equipped with four channels (405, 488, 568, and 647 nm), a 40x 1.3 and 60x NA 1.4 oil immersion objective and a C11440 ORCA-Flash4-oIT digital camera.

Higher resolution 3D images were taken in different channels by sequentially scanning using a laser scanning confocal microscope (Leica TCS SP8). The confocal microscope was equipped with a Diode 405/504 nm and Argon lasers (405, 488, 552, 638 nm). The images were taken by using the HP PL APO 63x/NA1.2 water immersion objective.

### Image Analyses

Image analyses were performed using ImageJ 1.53c or ICY.^1^

**FIGURE 1:**
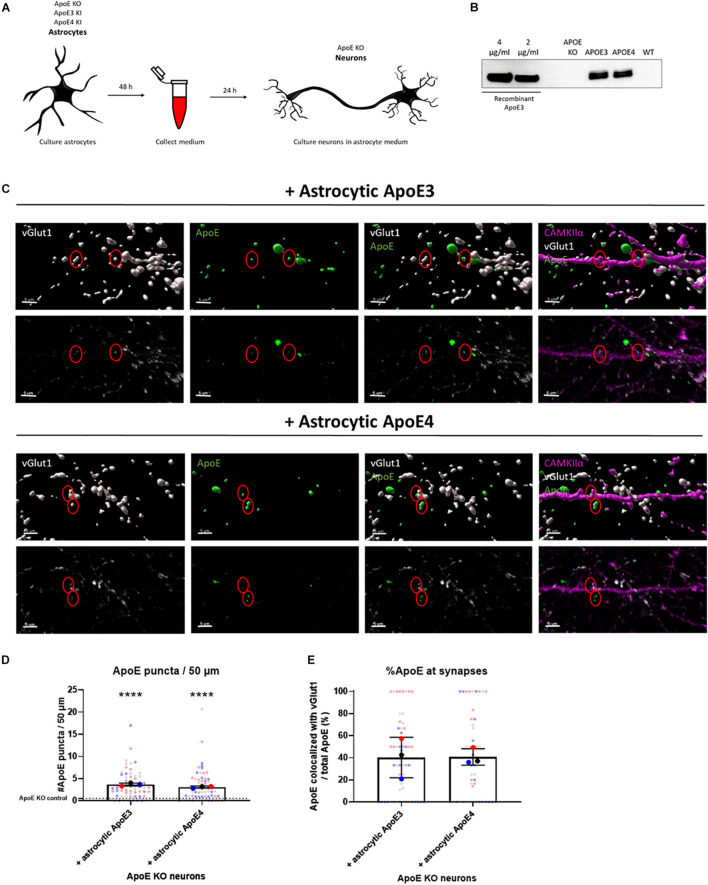
Astrocytic ApoE is detected at synaptic terminals in ApoE KO neurons cultured in ApoE3 and ApoE4 astrocyte conditioned medium for 24 h. **(A)** Schematic overview of astrocyte conditioned medium collection and treatment of ApoE KO primary neuron cultures. **(B)** Representative Western blot analysis of ApoE protein levels in conditioned medium from human ApoE3 and ApoE4 mouse astrocytes after 48 h. ApoE KO- and wild-type (WT) conditioned medium were added as negative controls. The different concentrations of recombinant ApoE3 give an indication of ApoE concentration in media. **(C)** Representative 3D reconstructed images (upper panels) and original confocal images (lower panels) of ApoE (green) and vGlut1 (gray) co-localization in ApoE KO neurons (19 DIV) cultured with ApoE3- and ApoE4-medium for 24 h. CAMKIIα (magenta) was used as a marker to identify glutamatergic neurons. Scale bar is 5 μm. A red circle indicates ApoE puncta at/close to vGlut1-postive synapses. **(D)** Quantification of ApoE puncta at/close to neurites analyzed using ICY spot detector (*N* = 3 embryos; *n* = 63 neurites per condition). The puncta detected in neurons cultured in astrocytic ApoE KO medium are indicated by a dotted line. ****p < 0.0001. **(E)** Quantification of the percentages of ApoE puncta co-localizing with vGlut1-positive presynaptic terminals using ICY colocalizer in relation to total ApoE puncta (*N* = 3 embryos; co-localization was analyzed in neurites containing ApoE puncta only, *n*_*ApoE3*_ = 55 neurites, *n*_*ApoE4*_ = 48 neurites). The data were shown in superplots to show the variation on both embryo and cell level. Data were expressed as mean ± SD. Statistical analyses were performed using Kruskal Wallis test followed by Dunn’s multiple comparisons test **(D)** or Mann Whitney test **(E)**. ****p < 0.0001.

The synaptic localization of ApoE was visualized using 3D reconstruction images. A 3D reconstruction of neurons labeled for CAMKIIα, ApoE and vGlut1 was created using Imaris (version 9.3) (Figures 1C, 3C). Surfaces were created for each individual channel. The ApoE surface settings in Imaris were corrected for ApoE KO neurons (Supplementary Figures 3A,6A).

For the quantification of ApoE puncta present at neurites or synaptic terminals (Figures 1D,E, 3D,E and Supplementary Figures 3B, 6B), 7 neurites per neuron were selected as individual regions of interest (ROI) using the area tool in ICY. The number of ApoE puncta present or in close proximity to neurites was quantified in ICY using spot detector. The settings used for detecting ApoE spots were corrected for non-specific spots detected in ApoE KO control neurons. The number and percentage of ApoE present at vGlut1-positive synaptic terminals was quantified using ICY colocalizer protocol.

Quantification of dendritic branching was performed of MAP2-labeled neurons using Sholl analysis using the Neuroanatomy ImageJ plugin (Ferreira et al., 2014; Figures 5A–C). Neurites from neighboring cells were carefully removed prior to analysis. Intersection points were automatically calculated in binary images with an increasing radius step of 30 μm and assessed by the Neuroanatomy ImageJ algorithm. Per embryo five different neurons were analyzed.

VGAT- and vGlut1-positive synaptic density was quantified per neuron using ImageJ (Figures 5E,F). The tracings of neurites were selected based on phalloidin staining using ImageJ plugin NeuroJ (Meijering et al., 2004) and synaptic density per 100 μm neurite length was determined based on VGAT-phalloidin and vGlut1-phalloidin combinations using the SynapCount v2 plugin (Mata et al., 2017). A total of five neurons for each embryo were analyzed.

### Live Cell Ca^2+^ Imaging

Primary neurons (18–20 DIV) were incubated with 3 μM Fluo-4 AM (Thermo Fisher Scientific, F14201), a Ca^2+^-indicator, for 30 min at 37°C prior to live cell imaging. The cultured mouse neurons were imaged by using an inverted Nikon Ti-E live cell microscope. The cells were imaged every 100 ms for a total duration of 2 min per field of focus. The primary neurons were kept in an incubator at 37°C containing 5% CO_2_ during the whole live cell imaging session.

### Live Cell Ca^2+^ Image Analysis

The Ca^2+^ recordings were analyzed using NETCAL software within MATLAB^2^ (Orlandi et al., 2017). Before the individual cells were analyzed, the recordings were corrected for drifting by averaging 10 frames each into one key frame, followed by standard preprocessing including subtract correction.

Each cell body was manually detected as a ROI. One ROI has a circle diameter between 6 and 10 pixels. After that, traces were extracted based on changes in fluorescent intensity over time. To reduce the noise of the traces, the data was normalized based on the deviation percentage from the original mean fluorescence (F0) using the following formula: 100 × (F–F0)/F0. The fluorescent traces were smoothed by applying a moving average filter of 5 frames wide. A spline division length, the length of each division used for spline fitting, of 15 s was used. The traces were corrected for small drifts by subtracting the spline after smoothing to the original spline. After smoothing of the traces, the smoothed traces were always compared to the original traces to avoid having abnormal traces or abrupt changes in baseline or the shape of the traces.

The ROIs were semi-automatically classified into three different groups: neurons, glia or silent cells, based on their fluorescent traces using AdaBoostM2 (Freund and Schapire, 1997) or RobustBoost (Freund, 2009), depending on the number of groups present in the culture (Supplementary Figure 5A). For each group representative traces were selected to let the software learn and classify the rest of the traces in the data. After the classification process, the traces were always refined to avoid false classification.

The classified neuron sub-group was further used to assess the neuronal properties at single cell level. For that a spike interference method was run using the OASIS algorithm (Friedrich et al., 2017) on the group classified as neurons. A lambda, i.e., a penalty parameter, of 5 was used. A thresholded interference method was used with model ar1. The minimal spike size constraint (S min) was 2.5 and the signal to noise parameter (Sn) was automatically estimated. In the spikes features given by the NETCAL program; a burst was defined as a connected group of spikes with intervals smaller than 1 s. Active neurons, defined as a neuron with more than 2 spikes per 2 min, were used for spike analysis.

### Statistical Analyses

Statistical analyses were performed using Graphpad Prism 8.4.1 software. The normal distribution of the data was assessed using Graphpad Prism or IBM SPSS statistics version 25 software prior to statistical testing by interpretation of histograms and normal QQ-plots of the data, and the Shapiro-Wilk test of normality. When the data was normally distributed and contained more than 2 independent groups, a one-way ANOVA was performed. If ANOVA showed a significant result, multiple comparisons were performed with Tukey correction to assess which groups were significantly different from each other. When the data was not normally distributed, non-parametric Mann Whitney (2 groups) or Kruskal-Wallis tests (>2 groups), followed by pairwise comparison testing using Dunn′s test, were used to test statistical significance between data sets. An alpha-level of ≤0.05 was considered significant. All data were expressed as mean ± SD unless stated otherwise. Violin plots were used to show the data distribution of the live cell Ca^2+^ experiments and the median and quartiles are indicated in these plots using dotted lines. The overall sample size (N) represents the number of independent embryos within an experiment, the small “n” indicates the total number of analyzed neurons or neurites within the embryos. The significant differences between different conditions were indicated as follows: ^∗^*p* < 0.05, ^∗∗^*p* < 0.01, ^∗∗∗^*p* < 0.001, ^****^p < 0.0001.

## Results

### Astrocytic ApoE Is Present in Neurites and Synapses

In the brain ApoE is mainly produced by astrocytes and transports lipids from astrocytes to other cells such as neurons. Thus, the majority of the ApoE present in the brain originates from astrocytes. To investigate whether ApoE produced by astrocytes binds and/or localizes to synaptic terminals, conditioned medium was collected from human ApoE3 KI, human ApoE4 KI and ApoE KO primary mouse astrocytes and then added to ApoE KO primary neurons (at 18–20 DIV) for 24 h (Figure 1A). ApoE KO primary neurons were used here to avoid the potential influence of endogenous mouse neuronal ApoE. Western blot analysis confirmed that abundant levels of human ApoE are secreted into ApoE3 KI and ApoE4 KI primary astrocyte media at similar concentrations (approximately 3 μg/ml), while ApoE KO astrocyte medium did not contain ApoE (Figure 1B and Supplementary Figure 1A). An ApoE antibody specifically binding to the lipid-binding domain of ApoE did not detect ApoE complexes in ApoE3 and ApoE4 astrocyte media under native conditions, while recombinant ApoE was detected, consistent with lipidated ApoE being present in astrocyte conditioned medium (Supplementary Figure 2).

24 h after culturing ApoE KO neurons in ApoE3 or ApoE4 astrocyte conditioned medium, both astrocytic ApoE3 and ApoE4 appear to co-localize with vGlut1-positive synaptic puncta (Figures 1C,E and Supplementary Figure 3), suggesting the presence of astrocytic ApoE at or near to vGlut1-positive pre-synaptic terminals, although more advanced microscopy techniques, such as super-resolution microscopy are required to more precisely specify a pre- or post-synaptic localization of added ApoE. Surface and intracellular labeling of ApoE show stronger ApoE labeling in permeabilized MAP2-positive neurites compared to non-permeabilized neurites indicated by MAP2-negative segments (Supplementary Figure 4). This suggests a primarly intracellular presence of ApoE in neurites and synapses. However, no significant difference in ApoE levels was detected at/or close to neurites and synapses between astrocytic ApoE3 and ApoE4 (Figures 1D,E and Supplementary Figure 3B), suggesting similar ApoE levels are present at or near synapses in the presence of astrocytic ApoE3 and ApoE4.

### Short but Not Long-Term Treatment With Astrocytic ApoE4 Increases Neuronal Excitability

**FIGURE 2:**
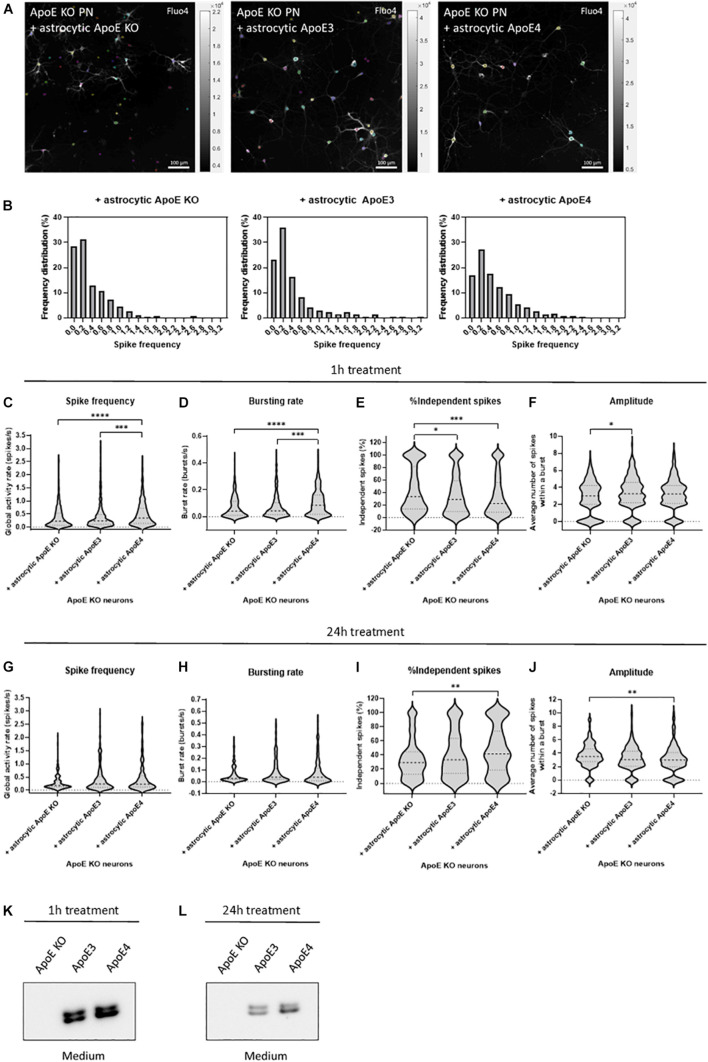
Astrocytic ApoE4 increases neuronal activity after treatment for 1 h, but not 24 h in ApoE KO primary neurons. **(A)** Representative images of ApoE KO neurons treated with astrocytic ApoE KO, ApoE3 or ApoE4 for 1 h (18–20 DIV) obtained by live cell Ca^2+^ imaging. The neurons were labeled for Fluo4 (white), a Ca^2+^ indicator. Each colored circle represents one region of interest (ROI). The scale bar represents 100 μm. **(B)** Representative frequency distribution plots of ApoE KO neurons cultured with ApoE KO, ApoE3 or ApoE4 astrocyte conditioned medium for 1 h. **(C–F)** Quantifications of spike frequency **(C)**, bursting rate **(D)**, percentages of independent spikes within a culture **(E)**, and amplitude **(F)** of ApoE KO neurons treated with ApoE KO, ApoE3 or ApoE4 astrocyte conditioned medium for 1 h analyzed using NETCAL program (*N*_*ApoE KO*_ = 12 embryos, *N*_*ApoE3*_ = 13 embryos, *N*_*ApoE4*_ = 14 embryos; *n*_*ApoE KO*_ = 291 neurons, *n*_*ApoE3*_ = 414 neurons, *n*_*ApoE4*_ = 426 neurons). **(G–J)** Quantification of spike frequency **(G)**, bursting rate **(H)**, percentage of independent spikes within a neuronal culture **(I)**, and amplitude **(J)** of ApoE KO, ApoE3 or ApoE4-medium treated ApoE KO neurons for 24 h (*N*_*ApoE KO*_ = 6 embryos, *N*_*ApoE3*_ = 6 embryos, *N*_*ApoE4*_ = 6 embryos; *n*_*ApoE KO*_ = 157 neurons, *n*_*ApoE3*_ = 173 neurons, *n*_*ApoE4*_ = 126 neurons). **(K,L)** Representative Western blot analysis of human ApoE proteins in neuronal medium of ApoE KO neurons treated with astrocytic ApoE KO, human ApoE3 or human ApoE4 medium for 1 h **(K)** or 24 h **(L)**. Data are expressed as violin plots. Kruskal Wallis tests in combination with Dunn’s multiple comparison tests were performed to detect statistical differences. *p < 0.05; **p < 0.01; ***p < 0.001; ****p < 0.0001.

Since astrocytic ApoE3 and ApoE4 were seen to localize at or near synapses, we asked whether astrocytic ApoE could influence spontaneous neuronal activity, and, more specifically, could induce neuronal hyperexcitability as previously shown in ApoE4 target replacement mice (Nuriel et al., 2017). To investigate this, ApoE KO primary neurons (18–20 DIV) were cultured in astrocyte conditioned medium collected from ApoE KO, ApoE3 KI and ApoE4 KI mouse primary astrocytes for 1 or 24 h (Figure 1A). Neuronal excitability was measured by live cell Ca^2+^ imaging using the calcium indicator Fluo-4, and individual cells were analyzed as ROI (Figure 2A). Individual ROI were sub-classified into neurons, glia or silent cells based on their characteristic Ca^2+^ tracing patterns (Supplementary Figure 5A), and active neurons were used for further analysis to specifically study neuronal activity.

After 1 h of culturing ApoE KO neurons in astrocyte conditioned medium, astrocytic ApoE4 induces increased global activity in neurons as detected by higher spike frequency and decreased inter-spike interval in ApoE4- compared to ApoE KO- and ApoE3-treated neurons (Figures 2B,C and Supplementary Figure 5C). While ApoE KO neurons cultured in ApoE KO or ApoE3 astrocyte media show a high proportion of neurons firing at a low spike frequency rate (Figure 2B), astrocytic ApoE4 treated ApoE KO neurons show a more evenly distributed firing rate per neuron, indicating increased highly active cells in the presence of acute (1 h) astrocytic ApoE4. Additionally, and in line with the increased firing rate, ApoE KO neurons treated with astrocytic ApoE4 also show an increased spontaneous bursting rate compared to ApoE KO neurons cultured with astrocytic ApoE KO or ApoE3 medium (Figure 2D and Supplementary Figure 5B). Further, astrocytic ApoE4-treated neurons induce a decreased proportion of independent spikes, i.e., spikes outside a burst, after 1 h of treatment compared to ApoE KO-treated neurons (Figure 2E). The proportion of independent spikes in a culture is an indicator of spike efficacy and represents isolated and not coordinated activity (Fernandez-Garcia et al., 2020). Reduced percentages of independent spikes within a culture negatively correlate with spike efficacy and network coordination. Our results suggest increased spike efficacy and network coordination in the acute presence of astrocytic ApoE4. Despite these changes detected in neuronal firing between astrocytic ApoE4 and astrocytic ApoE3, no alterations in number of active neurons within a culture were observed (Supplementary Figure 5D). Moreover, no significant differences in amplitude are seen in neurons cultured for 1 h with astrocytic ApoE4 compared to ApoE KO or ApoE3 media, while significantly decreased amplitudes are seen in neurons cultured in astrocytic ApoE KO compared to ApoE3 media (Figure 2F).

Under physiological conditions, astrocytes continuously produce ApoE in the brain. Therefore, we wanted to assess whether the observed acute effect on neuronal excitability induced by astrocytic ApoE4 was also detected after culturing ApoE KO neurons with ApoE3, ApoE4 and ApoE KO astrocyte medium for 24 h. However, after 24 h no significant differences in spike frequency, bursting rate, inter-spike interval and proportion of active neurons were seen between ApoE KO neurons treated with astrocytic ApoE KO, ApoE3 or ApoE4 media (Figures 2G,H and Supplementary Figures 5E,F). Interestingly and opposite to the 1 h treatment with astrocytic ApoE4, culturing ApoE KO neurons with astrocytic ApoE4 for 24 h significantly induces increased number of independent spikes per cell compared to ApoE KO neurons cultured in ApoE KO astrocyte medium (Figure 2I), suggesting a reduced synaptic efficacy in the presence of astrocytic ApoE4 after 24 h. A different effect on amplitude is also observed after 24 h of astrocytic ApoE4 media-treatment with neurons showing lower amplitudes compared to astrocytic ApoE KO media-treated neurons (Figure 2J).

To test whether these different effects in neuronal activity caused by astrocytic ApoE4 after 24 h of treatment with astrocyte medium might be caused by the loss of ApoE in the medium after longer time points, Western blot analysis of ApoE in neuronal medium was performed in ApoE KO neurons treated with ApoE KO, ApoE3 and ApoE4 astrocyte medium for 1 and 24 h (Figures 2K,L). Although the ApoE levels in media after 24 h are considerably reduced compared to after 1 h of treatment (about 72% reduction), considerable ApoE levels could still be detected after 24 h in neuronal medium from ApoE KO neurons treated with media collected from ApoE3 KI and ApoE4 KI astrocytes (Figure 2L). This suggests that the comparable neuronal activity seen in neurons treated with ApoE3 and ApoE4 astrocyte media for 24 h are not caused by a complete lack of ApoE in the medium. Altogether, after acute treatment, astrocytic ApoE4 induces enhanced neuronal activity in ApoE KO neurons. This increased effect on neuronal activity was not observed after longer-term culturing in the presence of astrocytic ApoE4.

### Endogenous Neuronal ApoE Is Detected at or Near Synaptic Terminals

**FIGURE 3:**
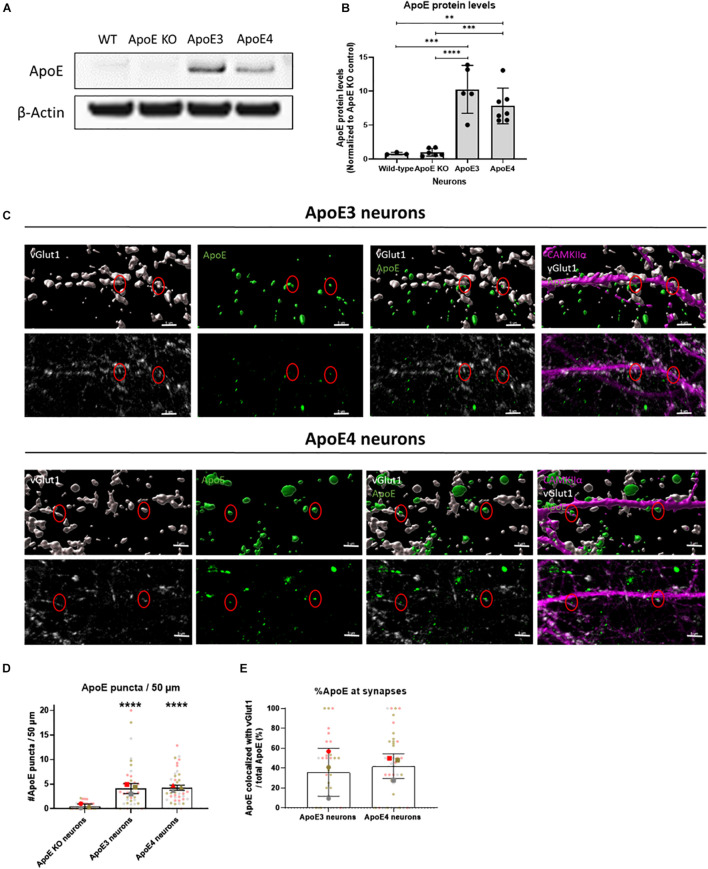
Endogenously expressed human ApoE in neurons can be detected at synaptic terminals. **(A)** Representative Western blot analysis of human ApoE protein levels in lysate of wild-type (WT), ApoE KO, ApoE3 KI and ApoE4 KI primary mouse neurons (15 DIV). β-Actin was used as a loading control. **(B)** Quantification of ApoE protein levels in WT, ApoE KO, ApoE3 KI and ApoE4 KI neuronal lysate detected by Western blot analysis (*N*_*wild–type*_ = 3 embryos, *N*_*ApoE KO*_ = 6 embryos, *N*_*ApoE3*_ = 5 embryos, *N*_*ApoE4*_ = 7 embryos). The data was normalized to ApoE protein levels in ApoE KO neurons (ApoE KO = 1). **(C)** Representative 3D Imaris reconstructed (upper panels) and non-reconstructed confocal images (lower panels) of ApoE (green), presynaptic marker vGlut1 (gray), and excitatory neuronal marker CAMKIIα (magenta) labeling in ApoE3 and ApoE4 primary neurons (19 DIV). Red circles indicate ApoE and vGlut1 co-localization. Scale bar is 5 μm. **(D,E)** Quantification of confocal microscopy shown in 3C of ApoE puncta in neurites per 50 μm (*N* = 3 embryos, *n* = 42 neurites per condition) **(D)** and percentages of ApoE puncta co-localizing to vGlut1-positive synaptic terminals (*N* = 3 embryos; neurites expressing ApoE are used in our co-localization analysis only, *n*_*ApoE3*_ = 33 neurites, *n*_*ApoE4*_ = 39 neurites) **(E)**. In panel **(D)**, significance levels compared to ApoE KO neurons are indicated in the graph. Statistical differences were assessed using Kruskal Wallis test, followed by Dunn’s test for multiple comparisons **(D)** or Mann Whitney test **(E)**. Data are shown in superplots and are expressed as mean ± SD. **p < 0.01; ***p < 0.001; ****p < 0.0001.

Under stress conditions, such as injury or aging, neurons have been shown to produce ApoE (Xu et al., 2006). Neuronal ApoE has previously been shown to play a critical role in GABAergic interneuron loss (Andrews-Zwilling et al., 2010) and thus might also be important in regulating neuronal activity in AD. Western blot analysis confirmed that human ApoE is present in ApoE3 and ApoE4 neuronal lysate and media (Figures 3A,B and Supplementary Figure 1B). No significant difference in intracellular ApoE protein levels was detected between neurons expressing the different human ApoE isoforms (Figure 3B). To study whether neuronal ApoE might also be present at synapses, the synaptic localization of neuronal ApoE was studied in ApoE KO, ApoE3 and ApoE4 neurons using confocal microscopy. In both ApoE3 and ApoE4 neurons ApoE puncta are detected at/close to neurites (Figures 3C,D and Supplementary Figure 6A). Co-localization analysis of ApoE with presynaptic marker vGlut1 revealed that around 40% of ApoE detected at neurites co-localizes with vGlut1-positive synapses in ApoE3 and ApoE4 neurons (Figures 3C,E and Supplementary Figure 3), suggesting also a synaptic localization of neuronal ApoE. No significant difference was found in the presence of ApoE at or near to synaptic terminals between the different ApoE isoforms.

### ApoE3 Neurons Show Increased Neuronal Activity Compared to ApoE4 Neurons

**FIGURE 4:**
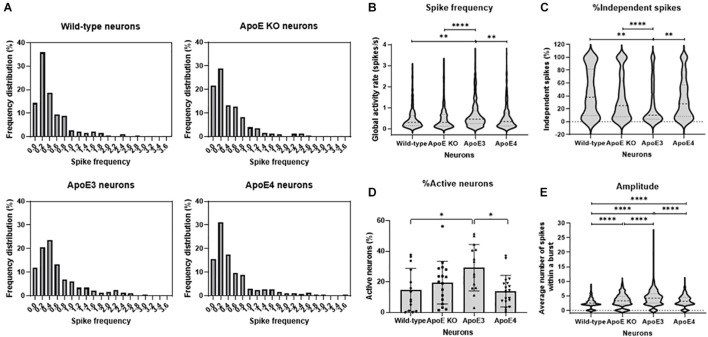
ApoE3 neurons show increased neuronal activity compared to wild-type, ApoE KO and ApoE4 neurons. **(A)** Representative frequency distribution graphs of wild-type, ApoE KO, ApoE3 and ApoE4 neurons (18–20 DIV) obtained using live cell Ca^2+^ imaging. **(B–E)** Quantifications of spike frequency **(B)**, percentage of independent spikes within a neuron culture **(C)**, percentage of active neurons, i.e., more than 2 spikes per 2 min, within a culture **(D)**, and amplitude **(E)** in wild-type, ApoE KO, ApoE3 and ApoE4 neurons (*N*_*wild–type*_ = 14 embryos, *N*_*ApoE KO*_ = 17 embryos, *N*_*ApoE3*_ = 14 embryos, *N*_*ApoE4*_ = 19 embryos; *n*_*wild–type*_ = 181 neurons, *n*_*ApoE KO*_ = 432 neurons, *n*_*ApoE3*_ = 474 neurons, *n*_*ApoE4*_ = 327 neurons). Data are expressed as violin plots **(B,C,E)** or mean ± SD **(D)**. Statistical analyses were performed using Kruskal-Wallis tests, followed by Dunn’s tests. *p < 0.05; **p < 0.01; ***p < 0.001; ****p < 0.0001.

Since neuronal ApoE, similar to ApoE secreted from astrocytes, appears to have a synaptic localization, live cell Ca^2+^ imaging was performed in wild-type, ApoE KO, human ApoE3 KI and human ApoE4 KI primary mouse neurons (18–20 DIV) to study whether neuronal ApoE also affects neuronal activity. In contrast to the effects of astrocytic ApoE on spontaneous neuronal activity, a more evenly distributed and on average higher spike frequency was observed in ApoE3 neurons compared to wild-type, ApoE KO and ApoE4 neurons (Figures 4A,B). In ApoE3 neurons a reduced proportion of independent spikes within a culture was observed compared to ApoE4, wild-type and ApoE KO neurons (Figure 4C), indicating increased synaptic efficacy of ApoE3 neuronal cultures. Surprisingly, while astrocytic ApoE did not alter the number of active neurons within a culture, neuronal ApoE3 showed a significantly increased proportion of active neurons compared to wild-type and ApoE4 neurons (Figure 4D). An isoform-dependent effect of ApoE on amplitude was also found with ApoE3 > ApoE4 = ApoE KO > wild-type neurons (Figure 4E). These results indicate a synaptic effect of neuronal ApoE, in particularly of ApoE3, on neuronal activity.

### Reduced Synaptic Density in ApoE4 KI Neurons

**FIGURE 5:**
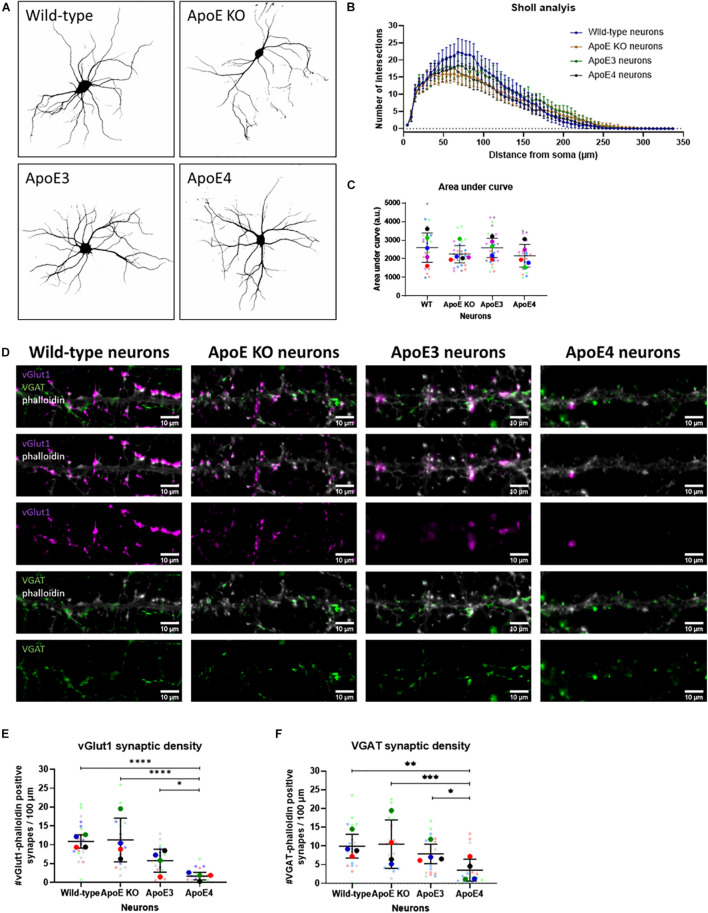
Neuronal ApoE4 reduces synaptic density, but not dendritic branching. **(A)** Representative binary images obtained by epifluorescence microscopy of wild-type, ApoE KO, ApoE3 and ApoE4 primary neurons (19 DIV). Neurons were labeled for neuronal/dendritic marker MAP2. **(B)** Graph showing Sholl analysis results of wild-type, ApoE KO, ApoE3 and ApoE4 neurons (*N* = 5 embryos per conditions, *n* = 25 neurons per condition). Data are shown as mean ± 95% CI. **(C)** Quantification of area under the curve of Sholl analysis data shown in panel **(B)** of wild-type, ApoE KO, ApoE3 and ApoE4 neurons (*N* = 5 embryos per condition, *n* = 25 neurons per condition). Statistical analysis was performed using one-way ANOVA. Data are shown in a superplot including mean ± SD of each condition. **(D)** Representative microscopy images of excitatory vGlut1-positive (magenta) and inhibitory VGAT-positive (green) presynapses in phalloidin-labeled (gray) neurons in wild-type, ApoE KO, ApoE3 and ApoE4 neurons (19 DIV). Scale bar represents 10 μm. **(E,F)** Quantification of vGlut1-phalloidin synaptic density **(E)** and VGAT-phalloidin synaptic density **(F)** in wild-type, ApoE KO, ApoE3 and ApoE4 neurons (*N* = 4 embryos per condition, *n* = 20 neurons per condition). Superplots represent all data points of each analyzed embryo and neuron. Data are shown as mean ± SD. Kruskall Wallis tests in combination with Dunn’s test were used to analyze statistical differences. *p < 0.05; **p < 0.01; ***p < 0.001; ****p < 0.0001.

To assess whether increased neuronal activity in ApoE3 compared to ApoE4 neurons might relate to changes in neuronal morphology and/or dendritic branching and complexity, wild-type, ApoE KO, ApoE3 and ApoE4 neurons (19 DIV) were studied using Sholl analysis, a method used to quantify dendritic intersections. Sholl analysis revealed no statistically significant differences in dendritic intersections and area under the curve, a measure of dendritic complexity, between the different neurons (Figures 5A–C), indicating no major changes in dendritic branching caused by the presence of different neuronal human ApoE isoforms.

ApoE KO, ApoE3 KI, ApoE4 KI, and wild-type neurons were further studied by assessing the density of excitatory and inhibitory synaptic inputs using microscopy of excitatory pre-synaptic marker vGlut1 and inhibitory pre-synaptic marker VGAT in ApoE KO, ApoE3, ApoE4, and wild-type primary neurons (19 DIV). Reduced pre-synaptic input was detected for both excitatory vGlut1-positive and inhibitory VGAT-positive synapses in ApoE4 neurons compared to the other neuron cultures (Figures 5D–F). These results suggest that the reduced neuronal excitability seen in ApoE4 neurons compared to ApoE3 neurons might be caused by reduced synaptic density.

### Neuronal Activity of AD Transgenic Neurons Increases in the Absence of ApoE and in the Presence of Astrocytic ApoE4 but Not ApoE3

**FIGURE 6:**
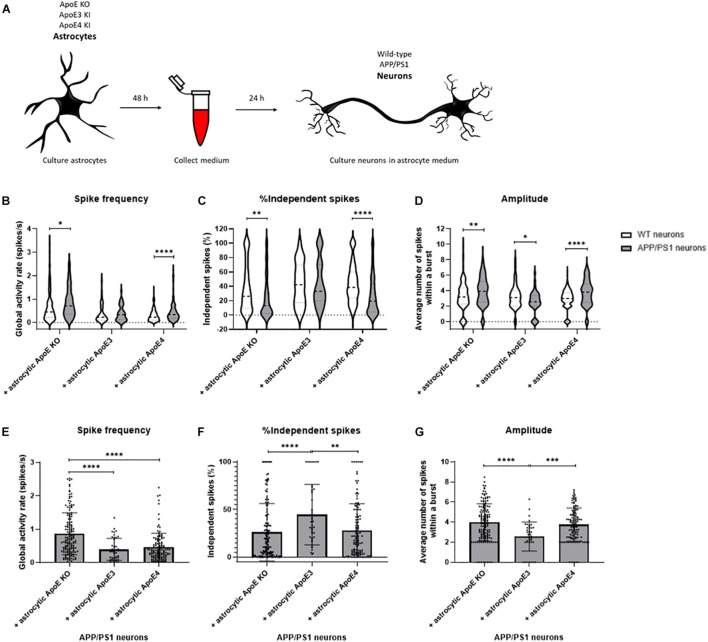
Neuronal activity is increased in APP/PS1 AD transgenic compared to wild-type neurons cultured in ApoE KO and ApoE4 astrocyte medium but not ApoE3 astrocyte medium. **(A)** Schematic overview of collection of ApoE KO, ApoE3 and ApoE4 astrocyte conditioned medium and subsequent treatment of wild-type and AD transgenic APP/PS1 neurons (18–20 DIV). **(B–D)** Quantification of spike frequency **(B)**, percentages of independent spikes per culture **(C)**, and amplitude **(D)** in wild-type and APP/PS1 neurons treated with ApoE KO, ApoE3 and ApoE4 astrocyte conditioned medium for 24 h (*N*_*wild–type* + *ApoE KO*_ = 5 embryos, *N*_*wild–type* + *ApoE3*_ = 4 embryos, *N*_*wild–type* + *ApoE4*_ = 4 embryos, *N*_*APP/PS1* + *ApoE KO*_ = 4 embryos, *N*_*APP/PS1* + *ApoE3*_ = 4 embryos, *N*_*APP/PS1* + *ApoE4*_ = 4 embryos; *n*_*wild–type* + *ApoE KO*_ = 119 neurons, *n*_*wild–type* + *ApoE3*_ = 48 neurons, *n*_*wild–type* + *ApoE4*_ = 171 neurons, *n*_*APP/PS1* + *ApoE KO*_ = 148 neurons, *n*_*APP/PS1* + *ApoE3*_ = 43 neurons, *n*_*APP/PS1* + *ApoE4*_ = 123 neurons). Data are expressed as violin plots. Statistical differences between wild-type and APP/PS1 neurons with different astrocyte medium conditions were analyzed using multiple *t*-tests. **(E–G)** Quantification of spike frequency **(E)**, percentages of independent spikes within a neuronal culture **(F)**, and amplitude **(G)** in APP/PS1 neurons treated with astrocytic ApoE KO, ApoE3 and ApoE4 astrocyte media for 24 h (*N*_*APP/PS1* + *ApoE KO*_ = 4 embryos, *N*_*APP/PS1* + *ApoE3*_ = 4 embryos, *N*_*APP/PS1* + *ApoE4*_ = 4 embryos; *n*_*APP/PS1* + *ApoE KO*_ = 148 neurons, *n*_*APP/PS1* + *ApoE3*_ = 43 neurons, *n*_*APP/PS1* + *ApoE4*_ = 123 neurons). Kruskal Wallis tests in combination with Dunn’s tests were performed to assess statistical differences between the different groups. Data are expressed as mean ± SD. *p < 0.05; **p < 0.01; ***p < 0.001; ****p < 0.0001.

As ApoE4 is known as the major genetic risk factor for AD and neuronal hyperexcitability has previously been described in AD mouse models overexpressing human Aβ (Busche et al., 2012, 2019), the effects of human ApoE on neuronal activity were studied in the presence of increased human Aβ. Similar to our approach described in Figure 1A, media were collected from ApoE KO, ApoE3 KI, and ApoE4 KI primary astrocyte cultures. Now, the media were used to treat wild-type and APP/PS1 mutant AD transgenic neurons for 24 h to study the effects of different human ApoE conditions on neuronal excitability in an AD mouse model (Figure 6A). APP/PS1 primary neurons in the absence of ApoE or in the presence of astrocytic ApoE4 media show significantly increased spike frequency and reduced proportion of independent spikes in comparison to wild-type neurons in the absence of astrocytic ApoE or in the presence of astrocytic ApoE4 media. Interestingly, these differences were not seen in APP/PS1 neurons cultured with ApoE3 astrocyte conditioned media (Figures 6B,C). APP/PS neurons treated with ApoE KO- and ApoE4-astrocyte conditioned media also have an increased spike amplitude compared to wild-type neurons, while a decrease in amplitude was observed in APP/PS1 compared to wild-type neurons in the presence of astrocytic ApoE3 (Figure 6D).

When specifically focusing on astrocytic ApoE effects on APP/PS1 neurons, astrocytic ApoE KO conditioned media significantly increases spike frequency compared to the presence of either astrocytic ApoE3 or ApoE4 (Figure 6E). Further, increased synaptic efficacy, indicated by a reduced proportion of independent spikes, was detected in APP/PS1 neurons cultured in the absence of ApoE and in the presence of astrocytic ApoE4, but not ApoE3 (Figure 6F). In addition, the amplitude was significantly increased in astrocytic ApoE KO- and ApoE4-treated APP/PS1 neurons, but not in APP/PS1 neurons treated with astrocytic ApoE3 media (Figure 6G). These results indicate a similar effect of astrocytic ApoE4 and the absence of ApoE on synaptic efficacy and amplitude in AD transgenic neurons.

## Discussion

Synaptic alterations and neuronal network dysfunction are some of the earliest changes in AD. Synaptic dysfunction and loss correlate strongly with cognitive impairment in AD, and ApoE4, the major genetic risk factor for AD, has been shown to be involved in synaptic function and neuronal network activity (Dumanis et al., 2009; Filippini et al., 2009; Chen et al., 2010; Kim et al., 2014; Nuriel et al., 2017). Previously, Lin et al. (2018) described specific changes induced by ApoE4 in different brain cell types. Here, we revealed a cell-type specific effect of astrocyte- and neuron-derived ApoE on neuronal activity *in vitro*. We provide evidence that astrocytic ApoE4, after acute administration, induces enhanced neuronal activity, whereas the presence of the ApoE3 isoform generated by neurons increases neuronal excitability. Additionally, we demonstrated that increased neuronal activity was induced in AD transgenic compared to wild-type neurons in the complete absence of ApoE or presence of astrocytic ApoE4, but not in the presence of astrocytic ApoE3.

ApoE4 has been described as playing a role in the regulation of synaptic plasticity and neuronal morphology (Nathan et al., 1994; Trommer et al., 2004; Dumanis et al., 2009; Chen et al., 2010). However, the presence of ApoE at synaptic terminals and whether it can induce a direct effect on synapses remain poorly understood. Our data support a synaptic localization of added and endogenous ApoE. Interestingly, the cellular source of ApoE, whether astrocyte- or neuron-derived, did not appear to alter the synaptic presence of ApoE, and both astrocyte- and neuron-produced ApoE were detected at or near synapses. Higher resolution imaging will be necessary to ascertain whether ApoE is more specifically pre- and/or post-synaptic. Although our study appears to be the first to describe synapse-associated ApoE by confocal microscopy upon adding ApoE to neurons, previous research has shown the presence of ApoE at synapsin-1-positive pre-synaptic terminals in brain using array tomography (Koffie et al., 2012). In addition, ApoE has been detected in synaptosomes from human brain (Arold et al., 2012), further emphasizing the apparent synaptic localization of ApoE. In our neuronal cultures, only a subset of all synapses contained ApoE. Koffie et al. (2012) estimated that 18–34% of all synapses in brain are positive for ApoE. Another study found that in AD post-mortem brains, 90% of the synapsotomes that are positive for ApoE receptors LRP1 or LDLR are also positive for ApoE, compared to 21% ApoE-positivity in the total synaptosome population (Bilousova et al., 2019).

Proper neuronal activity and network function are critical for learning and memory. Prior work has shown that the ApoE4 genotype could induce neuronal hyperactivity in both mouse models and humans (Filippini et al., 2009; Klein et al., 2014; Nuriel et al., 2017). Here, we found that acute ApoE4 astrocyte conditioned media induced increased firing frequency and synaptic efficacy, which further supports ApoE4′s effect on enhancing neuronal activity. Moreover, the ApoE4 gene is associated with a higher risk and earlier age of onset of temporal epilepsy (Gouras et al., 1997; Briellmann et al., 2000; Salzmann et al., 2008), linking ApoE4 to dysregulated neuronal firing. The underlying cellular mechanisms leading to ApoE4-induced hyperexcitability remain poorly understood. It was reported that preventing hyperexcitability improved memory performances in AD transgenic mice (Kleen et al., 2010; Kazim et al., 2017; Fischer et al., 2020). Interestingly, Ca^2+^-induced hyperactivity was recently noted as occurring in ApoE4 astrocytes, suggesting that altered neuronal activity might also be influenced by changes in astrocyte excitability (Larramona-Arcas et al., 2020).

Remarkably, although an astrocytic ApoE4-induced increase in neuronal activity was found after 1 h treatment, after 24 h this effect disappeared. ApoE can be degraded in media by proteases (Tamboli et al., 2014), however, as shown in Figure 2L, considerable ApoE protein levels are still detected in media after culturing with neurons for 24 h, suggesting that the lack of ApoE-induced effect on neuronal activity after 24 h is not caused by an absence of ApoE from degradation. In this study we showed that acute astrocytic ApoE4 induced neuronal hyperactivity. However, prolonged periods of enhanced activity could result in energy depletion and impaired mitochondrial function, and therefore a potential explanation for the lack of ApoE4 effect after longer time points might be energy depletion from neurons caused by impaired mitochondrial function (Ioannou et al., 2019). Hyperactive neurons increase levels of toxic free fatty acids, but neurons have low capacity to oxidize these toxic fatty acids into energy using mitochondria (Schonfeld and Reiser, 2013; Ioannou et al., 2019). In the presence of astrocytes, toxic free fatty acids from neurons can be transferred via ApoE to astrocytes, where they are stored in lipid droplets or oxidized (Belanger and Magistretti, 2009; Ioannou et al., 2019). However, because of the low abundance of astrocytes in our cultures, toxic free fatty acids might not be removed from neurons efficiently, potentially causing lack of energy sources and mitochondrial dysfunction after chronic hyperactivity. Alternatively, the lack of ApoE4-induced effect on neuronal activity after 24 h could potentially be explained by a negative feedback loop reducing synaptic activity triggered after chronic enhanced neuronal firing by homeostatic synaptic downscaling (Siddoway et al., 2014).

An imbalance between excitatory and inhibitory inputs can potentially lead to hyperexcitability caused by ApoE4 (Najm et al., 2019). For example, an imbalance in excitatory/inhibitory synapses can lead to hyperexcitability in the hippocampus (Bateup et al., 2013). Furthermore, a study by Nuriel et al. (2017) suggested that ApoE4-induced hyperexcitability can be caused by loss of inhibitory tone. Likewise, inhibitory GABAergic interneuron loss has been associated with ApoE4 genotype (Knoferle et al., 2014). Interestingly, GABAergic interneuron loss seems to be associated with the ApoE produced by neurons, rather than astrocytes (Knoferle et al., 2014). Because of this proposed role of neuronal ApoE on neuronal activity, in the current study we examined neuronal activity in different ApoE-expressing neurons. Hyperexcitability was not seen in our cultured neurons expressing human ApoE4. However, ApoE3 neurons appeared to have increased neuronal activity compared to ApoE4. ApoE4 is associated with reduced synaptic regulation and synaptic plasticity, compared to ApoE3 (Trommer et al., 2004; Chen et al., 2010). Such uncontrolled regulation of synaptic strength might deleteriously affect learning and memory (Garcia-Alvarez et al., 2015). Although most studies support ApoE4-induced hyperexcitability, conflicting data have also been published showing, for example, reduced neuronal activity in ApoE4 mice (Wang et al., 2005). These apparently contradictory data might possibly be explained by differences in brain regions examined or age of mice. Of note, one significant difference between our astrocytic and neuronal ApoE experiments is that only the neuronal cultures contained human ApoE continuously also during development. Therefore, changes caused by the ApoE genotype during development might underlie differences in neuronal activity. In this study, we could not observe significant differences in dendritic morphology between the different ApoE neuronal cultures. However, both excitatory and inhibitory pre-synaptic inputs were decreased in ApoE4 neurons compared to wild-type, ApoE KO and ApoE3 neurons. Previous literature described reduced neuronal outgrowth in ApoE4 compared to ApoE3 neurons (Nathan et al., 1994, 2002). In addition, synaptic density, in line with our data, has been shown to be reduced in ApoE4 neurons (Dumanis et al., 2009; Nwabuisi-Heath et al., 2014).

ApoE4 has been shown to induce detrimental effects in different cell types of the brain (Lin et al., 2018). The mechanisms and cell types associated with ApoE4 that are most crucial to AD remain unknown. It is important to be aware that the majority of ApoE in the brain is produced by astrocytes (Boschert et al., 1999), therefore effects of ApoE4 on neuronal activity observed *in vivo* in mice or in humans is most likely caused by astrocytic ApoE. Additionally, microglia can also generate ApoE (Xu et al., 2006), particularly activated microglia, and could therefore also potentially contribute to the ApoE4-induced effects seen in AD. However, a role of neuronal ApoE, that is generated during injury and stress such as aging (Boschert et al., 1999; Xu et al., 2006), might be quite important in AD.

Aging and ApoE4 are major risk factors for AD. An increased incidence of epileptic seizures is detected in MCI and early AD patients compared to healthy individuals (Vossel et al., 2013). Busche et al. (2019) previously described neuronal hyperactivity in APP/PS1 neurons overexpressing human Aβ. In this study, Ca^2+^ imaging revealed that APP/PS1 neurons, in the absence of ApoE, show neuronal hyperexcitability compared to wild-type neurons. Enhanced neuronal activity is also seen in APP/PS1 neurons cultured with astrocytic ApoE4, but interestingly, not with ApoE3. This suggests a potential protective mechanism of astrocytic ApoE3 on neuronal hyperexcitability in AD.

In conclusion, our study proposes a cell source specific effect of ApoE whereby astrocytic and neuronal ApoE induce different effects on neuronal activity. In addition, astrocytic ApoE3 potentially induces a protective effect in AD transgenic neurons by blocking enhanced neuronal activity. Further research is needed to elucidate the underlying cellular mechanisms of astrocytic and neuronal ApoE on neuronal excitability in both AD and non-AD conditions. Our findings contribute to the better understanding of ApoE, in particular the major genetic risk factor for AD ApoE4, in synaptic and neuronal network dysfunction during early stages of AD and may help provide new insights for future AD therapeutics based on ApoE4 and neuronal dysfunction.

## Data Availability Statement

The raw data supporting the conclusions of this article will be made available by the authors, without undue reservation.

## Ethics Statement

The animal study was reviewed and approved by Malmö/Lund Ethics Committee on Animal Testing, Lund University.

## Author Contributions

SK and GG conceived the work. SK wrote original draft and acquired the data. SK, LT-G, IM, and GG analyzed data and reviewed and edited the manuscript. GG acquired funding. All authors contributed to the article and approved the submitted version.

## Conflict of Interest

The authors declare that the research was conducted in the absence of any commercial or financial relationships that could be construed as a potential conflict of interest.

## Publisher’s Note

All claims expressed in this article are solely those of the authors and do not necessarily represent those of their affiliated organizations, or those of the publisher, the editors and the reviewers. Any product that may be evaluated in this article, or claim that may be made by its manufacturer, is not guaranteed or endorsed by the publisher.

## Footnotes

1
www.icy.bioimageanalysis.org

2
http://www.itsnetcal.com/
