# Supplementary material for: Astrocytic and Neuronal Apolipoprotein E Isoforms Differentially Affect Neuronal Excitability
Source: Front Neurosci. 2021 Sep 21;15:734001. doi: 10.3389/fnins.2021.734001 (PMC8490647; doi:10.3389/fnins.2021.734001)
Supplement: Supplementary file 1 [file Image_1.pdf]

Supplementary Material

Supplementary figure 1

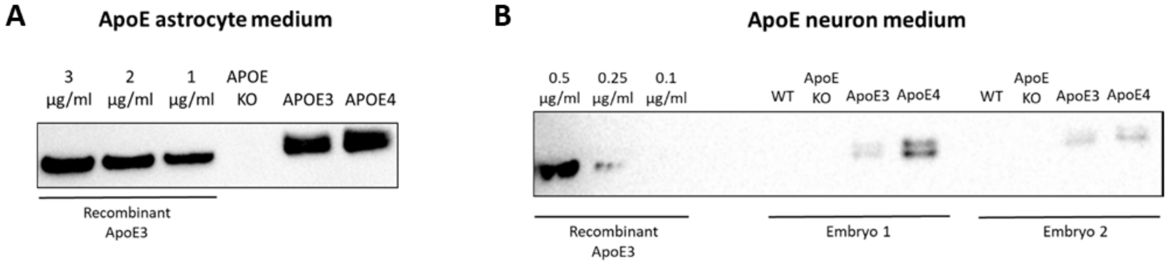

Supplementary Figure 1: Estimation of ApoE concentration in astrocyte and neuron media. A. Representative Western blot of ApoE protein levels in astrocyte conditioned media collected from ApoE KO, ApoE3 or ApoE4 primary astrocytes. Different recombinant ApoE3 concentrations were loaded on the gel to be able to estimate the protein concentration of ApoE in media. **B.** Representative Western blot of ApoE in wild-type (WT), ApoE KO, ApoE3 and ApoE4 neuron media from two independent cultures. To estimate the ApoE concentration in neuron media, lower concentrations of recombinant ApoE3 were used.

Supplementary figure 2

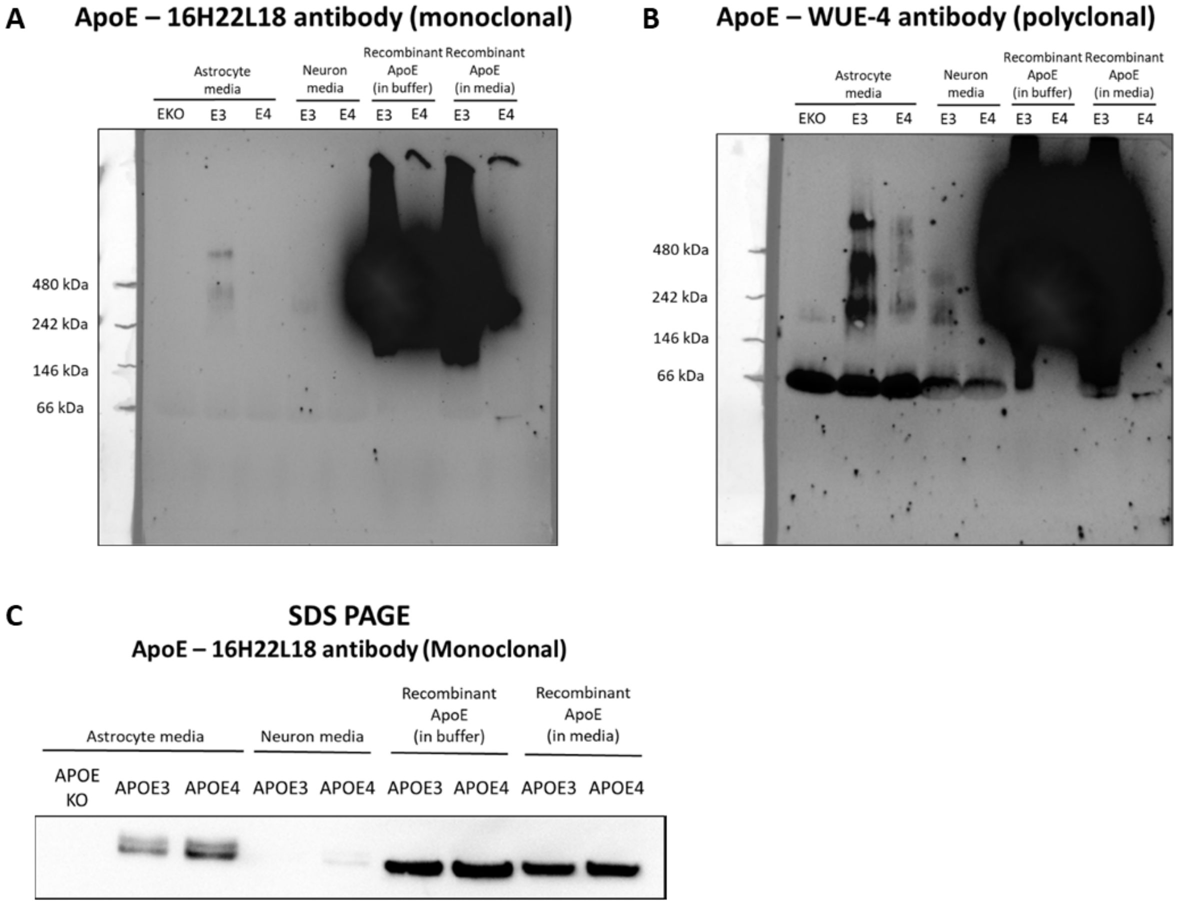

Supplementary Figure 2: Native structures of cell-produced and recombinant ApoE under non-denaturing conditions. A-B. Representative Blue-Native PAGE of ApoE complexes in ApoE KO (EKO), ApoE3 (E3) and ApoE4 (E4) astrocyte medium; ApoE3 and ApoE4 neuron medium; and recombinant ApoE3 and ApoE4 using monoclononal 16H22L18 ApoE antibody **(A)** and polycloncal WUE-4 ApoE antibody **(B)**. Recombinant ApoE was added to the Blue-Native PAGE gel in a concentration of 2.5 µg/ml and incubated in either 0.1% BSA in milli-Q water or incubated in warm complete neurobasal media for 20 min prior to loading. **C.** Representative SDS PAGE of ApoE protein levels in the same media and recombinant ApoE samples as used in Supplementary Figure 2A-B, ApoE antibody 16H22L18 was used to detect ApoE.

Supplementary figure 3

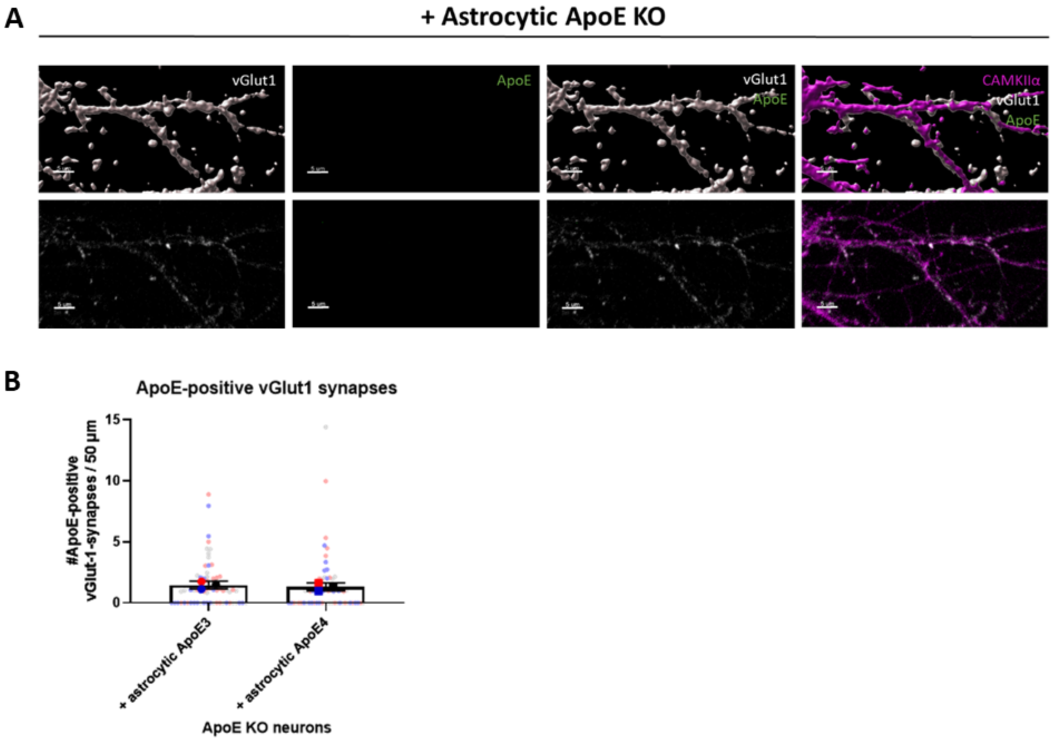

Supplementary Figure 3: No ApoE is detected in ApoE KO neurons treated with ApoE KO astrocyte conditioned media. A. Representative 3D reconstruction images (upper panel) and original confocal images (lower panel) of ApoE KO neurons treated with ApoE KO astrocyte medium for 24 hours (19 DIV). The neurons are labelled for presynaptic marker vGlut1 (grey), ApoE (green) and excitatory neuron marker CAMKIIα (magenta). Scale bar represents 5 µm. **B.** Quantification of the absolute number of ApoE puncta co-localizing to vGlut1-positive synaptic terminals per 50 µm neurite in ApoE KO neurons treated with ApoE3 or ApoE4 astrocyte medium for 24 hours (N = 3 embryos; n = 63 neurites per conditions). Data are expressed as mean ± SD.

Supplementary figure 4

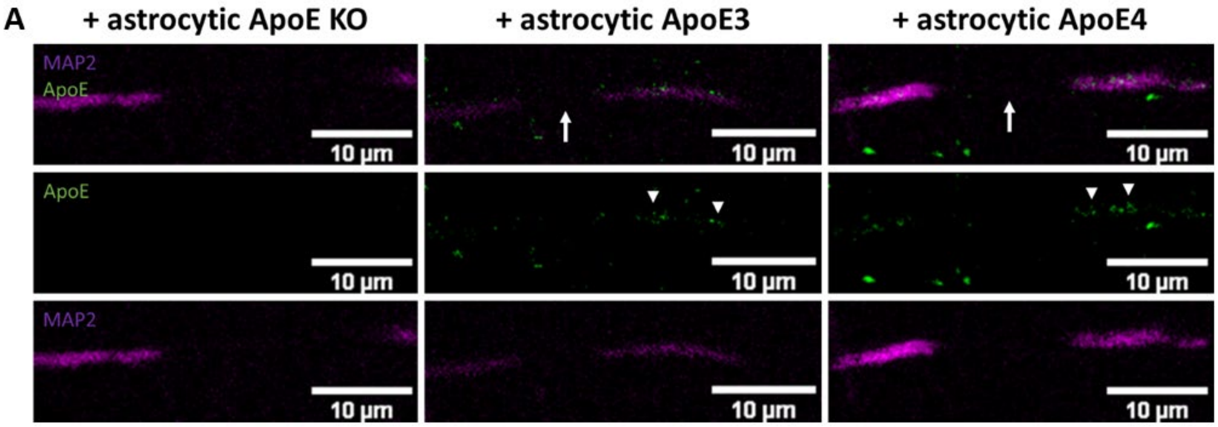

Supplementary Figure 4: Intracellular and surface labeling of ApoE in ApoE KO neurons treated with astrocyte media. A. Representative confocal microscope images of non-permeabilized ApoE KO neurons treated with astrocytic ApoE KO, ApoE3 or ApoE4 media for 4 hours. The neurons were non-permeabilized, but PFA-fixed, resulting in partial membrane permeabilization caused by PFA. Permeabilized sections of the neurite, positive for MAP2 labeling (magenta), show intracellular ApoE labeling (green). Non-permeabilized sections, negative for MAP2 labeling, indicate surface labeling of ApoE. Note the more evident labelling of ApoE3 and ApoE4 in the permeabilized segments (MAP2-positive; arrowheads) compared to non-permeabilized (MAP2-negative; arrows) segments of dendrites. Scale bar is 10 µm.

Supplementary figure 5

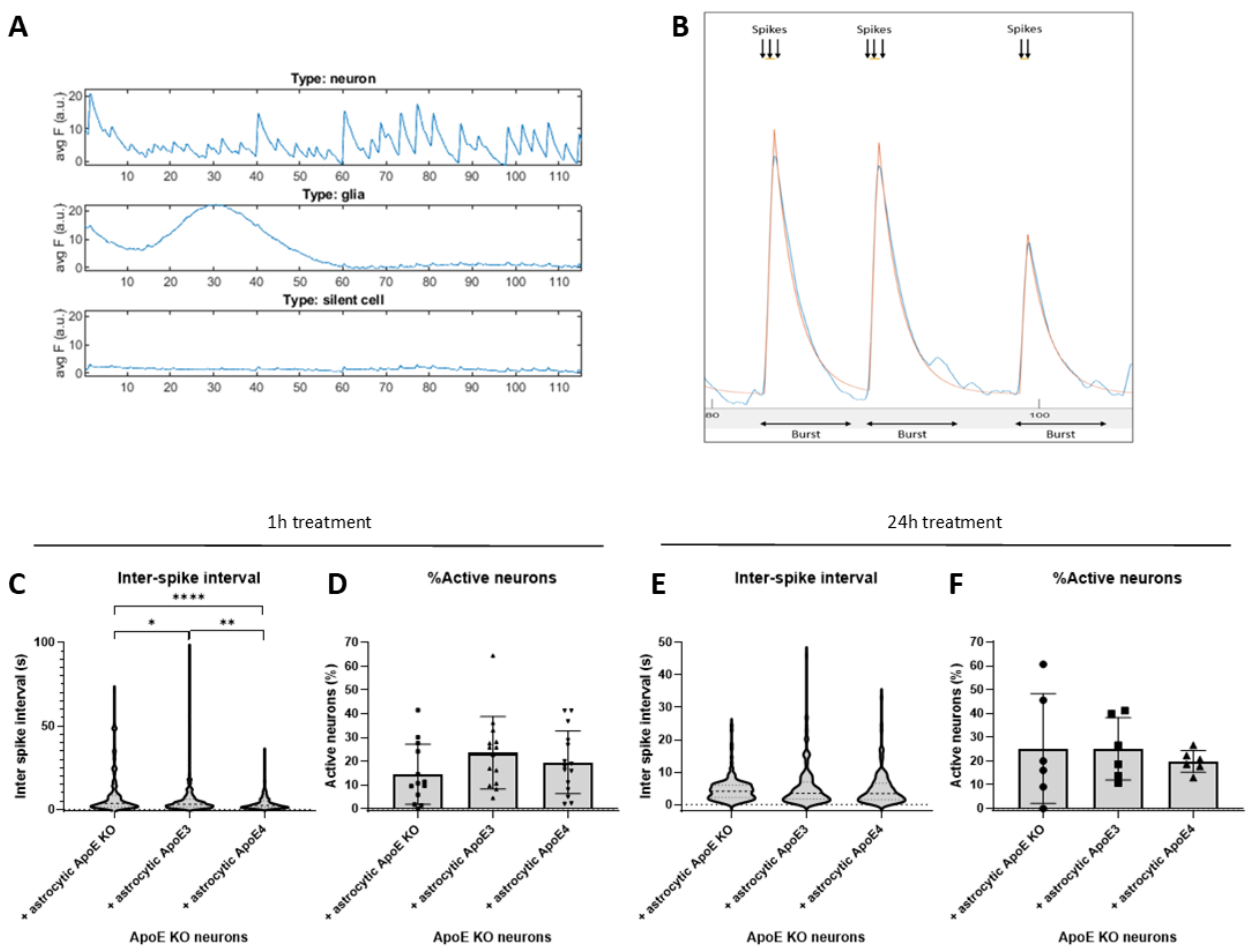

Supplementary Figure 5: Astrocytic ApoE4 reduces inter-spike intervals after 1 hour treatment of ApoE KO neurons. A. Representative fluorescent traces, representing changes in fluorescent intensity of Fluo-4 over time, of neurons (upper panel), glia (middle panel) and silent cells (lower panel). The fluorescent traces were used to classify neurons (upper panel), glia (middle panel) and silent cells (lower panel) into sub-groups based on their tracing pattern using the NETCAL software. **B.** Clarifying image of spike bursts to establish the definition of a single burst and spike. A spike is indicated by an arrow; a burst is shown by a double sided arrow. **C-D.** Quantification of inter-spike intervals ($N_{ApoE\ KO}$ = 12 embryos, N_{ApoE3} = 13 embryos, N_{ApoE4} = 14 embryos; $n_{ApoE\ KO}$ = 291 neurons, n_{ApoE3} = 414 neurons, n_{ApoE4} = 426 neurons) **(C)** and percentages of active neurons within a culture ($N_{ApoE\ KO}$ = 12 embryos, N_{ApoE3} = 13 embryos, N_{ApoE4} = 14 embryos) **(D)** in ApoE KO neurons treated with ApoE KO, ApoE3 or ApoE4 astrocyte medium for 1 hour. **E-F.** Quantification of inter-spike intervals ($N_{ApoE\ KO}$ = 6 embryos, N_{ApoE3} = 6 embryos, N_{ApoE4} = 6 embryos; $n_{ApoE\ KO}$ = 157 neurons, n_{ApoE3} = 173 neurons, n_{ApoE4} = 126 neurons) **(E)** and percentages of active neurons per culture ($N_{ApoE\ KO}$ = 6 embryos, N_{ApoE3} = 6 embryos, N_{ApoE4} = 6 embryos) **(F)** in ApoE neurons cultured in ApoE KO, ApoE3 or ApoE4 astrocyte medium for 24 hours. Data are shown as violin plots **(C, E)** or mean ± SD **(D, F)**. *$p < 0.05$, **$p < 0.01$, ****$p < 0.0001$.

Supplementary figure 6

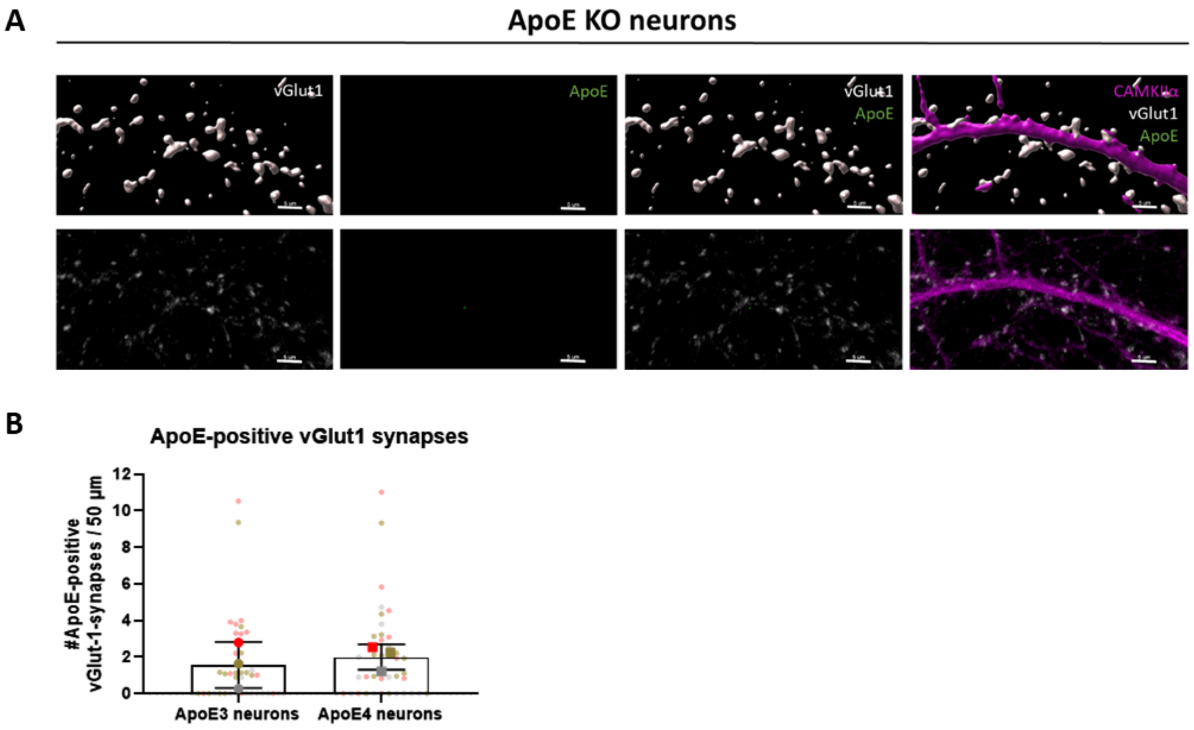

Supplementary Figure 6: ApoE KO control neurons do not express human ApoE. A. Representative 3D reconstructed (upper panel) and confocal images (lower panel) of ApoE (green), vGlut1 (grey) and CAMKIIα (magenta) labelled ApoE KO neurons (19 DIV). Scale bar is 5 µm. **B.** Quantification of absolute number of ApoE and vGlut1 co-localized puncta per 50 µm in ApoE3 and ApoE4 primary neurons (19 DIV) (N = 3 embryos, n = 42 neurites per condition). Data are expressed as mean ± SD.
